# Screening, identification and interaction analysis of key MicroRNAs and genes in Asthenozoospermia

**DOI:** 10.7150/ijms.54460

**Published:** 2021-02-06

**Authors:** Liman Li, Song Chen

**Affiliations:** 1 Center for Translational Medicine, Key Laboratory of Birth Defects and Related Diseases of Women and Children (Sichuan University), Ministry of Education, West China Second University Hospital, Sichuan University, Chengdu, China.; 2 Department of Cardiovascular Surgery, Zhongnan Hospital of Wuhan University, Wuhan, China.

**Keywords:** Asthenozoospermia, miRNAs, genes

## Abstract

**Background:** Asthenozoospermia, one of the most common causes of male infertility, is a complicate multifactorial pathological condition that genetic factors are involved in. However, the epigenetic signature and mechanism of asthenozoospermia still remain limited. Our study aimed to confirm the key microRNAs (miRNAs) and genes in asthenozoospermia and demonstrate the underlying epigenetic regulatory mechanisms.

**Methods:** We screened out and pooled previous studies to extracted potential differentially expressed miRNAs (DEMs). GSE22331 and a published profile dataset were integrated to identify differentially expressed genes (DEGs). Pathway and gene ontology analysis were performed using DAVID. A protein-protein network (PPI) was constructed using STRING. The target genes of DEMs were predicted using TargetScan and the miRNA-mRNA network was built.

**Results:** We reported 3 DEMs and 423 DEGs by pooling included dataset and published studies. Pathway analysis showed that these DEGs might participate in signaling pathways regulating pluripotency of stem cells, Wnt signaling pathway and Notch signaling pathway. 25 hub genes were identified, and the most significant gene was BDNF. We screened out the overlapped DEGs between the predicted target genes of 3 DEMs and the 423 DEGs. Finally, a potential miRNA-mRNA regulatory network was constructed.

**Conclusion:** This study firstly pooled several published studies and a GEO dataset to determine the significance of potential miRNAs and genes, such as miR-374b, miR-193a, miR-34b, BDNF, NTRK2, HNRNPD and EFTUD2 in regulating asthenozoospermia and underscore their interactions in the pathophysiological mechanism. Our results provided theoretical basis and new clues for potential therapeutic treatment in asthenozoospermia. Validations *in vivo* and *in vitro* are required in future studies.

## Introduction

**Figure 1:**
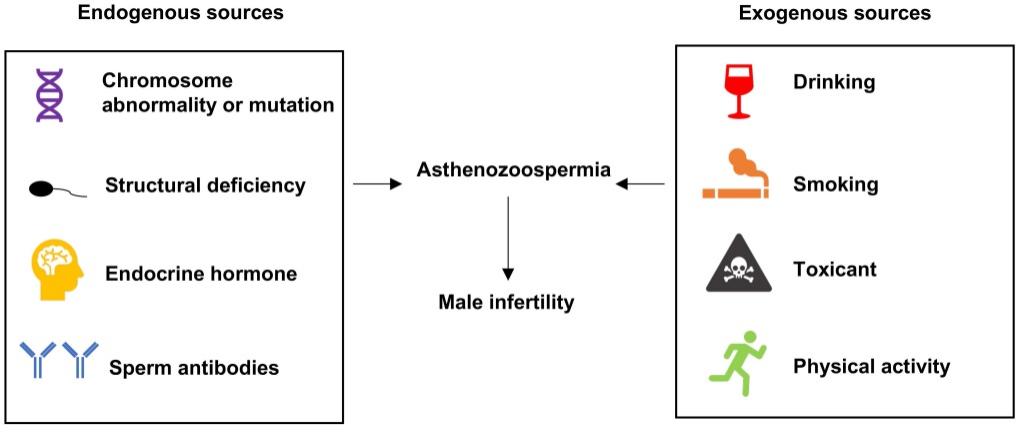
Sources leading to asthenozoospermia.

Male infertility is a severe problem in human reproduction health 1, 2. Abnormal sperm morphology, poor sperm motility and low sperm count are identified as common causes of male infertility. According to the criteria reported by the World Health Organization, asthenozoospermia is characterized by total motility < 40% and progressive motility < 32% in fresh semen samples 3. Several factors have been confirmed to be associated with asthenozoospermia, as shown in **Figure 1**. Chromosomal abnormality was correlated with sperm deficiency including asthenozoospermia 4. The multiple morphological abnormalities of the sperm flagella (MMAF) is one of the most known causes of asthenozoospermia. Mutations in the mitochondrial genome have been reported in MMAF, such as DNAH1, CFAP43, CFAP44, CFAP69, and QRICH2 5-8, and study have shown compelling evidence that the loss-of-function mutations of these genes could induce MMAF and lead to male infertility. Hormone could also modulate spermatogenesis and impact fertilization. In a clinical trial, most of 786 subfertility patients were characterized as idiopathic oligo or asthenozoospermia who improved their semen quality and concentration by receiving hormonal treatment 9. Sperm motility was under the control of flagellum 10 and any structural or functional defects in flagellum would lead to reduced motility and induce asthenozoospermia 11. Moreover, the anti-sperm antibodies played key roles in female and male immunological infertility and sperm-immobilizing antibodies that bound to the surface of ejaculated sperm could impact the sperm motility 12. There were other exogenous sources could result in asthenozoospermia, for example, drinking alcohol, smoking, toxicant and physical activity also have effect on the quality of sperm 13-17. However, the epigenetic signatures which affect asthenozoospermia are not well understood and require to be further investigated.

Currently, increasing genetic factors contributing to male infertility were reported. Alterations were observed between fertile men and asthenozoospermia patients' transcriptomes, including pathways and genes which were associated with this disease. SEMG1 mRNA and protein expression were upregulated in asthenozoospermia patients compared with that in normozoospermia group 18. CABYR and ROPN1 mRNA were significantly downregulated in asthenozoospermia patients' samples and a positive correlation was confirmed between the expression of the two genes, indicating that the co-expression of CABYR and ROPN1 was a prerequisite for normal sperm motility and flagellar function 19. In addition, miRNAs also have dysregulated expressions in testicular biopsy samples and sperms of infertile men. The expression of miR-34b in the control group was significantly higher than that in the asthenozoospermia group, demonstrating that miR-34b might serve as a novel biomarker of male subfertility 20. The downregulated expression of miR-525 and upregulated expression of miR-151a were also associated with asthenozoospermia and male infertility 18, 21.

In this study, we explored the differential expressed miRNAs (DEMs) and differentially expressed genes (DEGs) in asthenozoospermia patients through reanalyzing the data from published studies and a GEO dataset. Then, we conducted pathway and gene ontology analysis of DEGs using DAVID. The protein-protein interaction (PPI) network was constructed by STRING and DEMs' target genes were predicted by TargetScan. Our results proposed a novel insight into the related biological characteristics and molecular pathways of the DEMs and DEGs in asthenozoospermia. A miRNA-mRNA network further exhibited the potential mechanisms linked to asthenozoospermia.

## Methods

### Literature search of DEMs

Firstly, we searched GEO database for published miRNA profiling data of ashenozoospermia, however, there was no available result. Then, we conducted a systematic search in PubMed and Web of Science and found 4 studies, which contained miRNA profiling results in ashenozoospermia 22-25. All of samples used in 4 studies were semen samples and all of them used normal fertile individuals as controls. All studies identified the alteration trends of miRNAs (upregulated or downregulated). In order to investigate the involvement of crucial miRNAs in the modulation of ashenozoospermia, we pooled these 4 studies, and the overlapped miRNAs were presented with Venn diagram, using an online tool, Draw Venn Diagram (http://bioinformatics.psb.ugent.be/webtools/Venn/). Next, we chose the miRNAs with consistent expression alteration trends in studies as DEMs in following analysis.

### Data processing of DEGs

Xiaoning Zhang's study investigated the expression profiles of long noncoding RNA (lncRNA) and mRNA in mammalian sperm. mRNA between the normozoospermic and asthenozoospermic groups with q-value (FDR) ≤ 0.001 and fold-change (FC) ≥ 2 (|log2FC| > 1) was considered significantly changed, and we reanalyzed the changed mRNA in our following study 26. Next, we searched GEO database for the available mRNA expression profile data using keywords “ashenozoospermia” and “Homo sapiens”, and the GSE22331 dataset was downloaded. GSE22331 had 2 groups from normozoospermic and asthenozoospermic men. Each group was pooled by 30 sperm samples to obtain enough total RNA. Then, we screened significantly changed mRNA between normozoospermia and asthenozoospermia groups based on FC ≥ 2. Since there was only 1 pooled sample in each group, the FDR could not be calculated. Next, we obtained the intersected elements of significantly changed mRNA between GSE22331 dataset and Xiaoning Zhang's study which were used as DEGs in following analysis.

### KEGG and GO enrichment analysis of DEGs

We utilized DAVID (https://david.ncifcrf.gov/), an online biological information database, to visualize the DEGs enrichment of biological processes (BP), cellular components (CC), molecular function (MF) and biological pathways (P value < 0.05). The results of gene ontology and pathway analysis of DEGs were showed with histograms.

### Protein-protein interaction (PPI) network and module analysis

We utilized an online common software, STRING database (https://string-db.org), to build PPI network of DEGs. Then Cytoscape (www.cytoscape.org), a public source bioinformatics software platform, was applied to visualize and analyze the molecular interaction networks. The plugin, Molecular Complex Detection (MCODE), was applied to identify the most dense and significant module in PPI network based on degree cut-off = 2, node score cut-off = 0.2, max depth = 100 and k-score = 2. Another plugin, cytoHubba, was applied for screening out hub genes based on degrees. We chose the top 25 genes as hub genes and ClueGO and CluePedia plugins were performed to visualize biologic processes and pathways of hub genes.

### Construction of miRNA-mRNA network

The putative target genes of DEMs were predicted by an online database, TargetScan (http://www.targetscan.org/mamm_31/), which was commonly used for miRNA target prediction. We obtained the intersection of predicted target genes and DEGs. Besides, the intersected genes of each miRNA-mRNA network should oppositely express with DEMs. The regulatory network of miRNA-mRNA was constructed by Cytoscape.

### Hub gene expression in GEPIA

GEPIA (Gene Expression Profiling Interactive Analysis) was an online website which analyzed RNA sequencing expression data of tumors and normal samples from the TCGA and the GTEx projects (http://gepia.cancer-pku.cn). In our study, the expressions of hub genes in testicular cancer patients and controls were obtained from GEPIA.

## Results

### DEMs in asthenozoospermia

**Figure 2:**
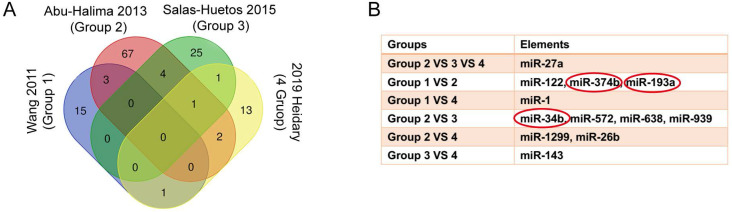
Explore the DEMs from 4 publications. (A) The Venn diagram showed the overlapped genes of 4 publications. (B) miR-374b, miR-193a and miR-34b had consistent expression alteration trends in studies and they were identified as DEMs in following analysis. DEMs, differentially expressed miRNAs.

We have searched 146 differentially expression miRNAs from 4 previous studies **(SI Table S1-4)**. Information about studies was shown in **Table 1**. By intersecting these 146 miRNAs of 4 studies **(Figure 2A)**, 12 overlapped miRNAs that might play critical roles in ashenozoospermia were selected. Among these 12 miRNAs, miR-374b, miR-193a and miR-34b were finally identified as DEMs because of their consistent expression trends in above studies **(Figure 2B)**.

### DEGs in asthenozoospermia

**Figure 3:**
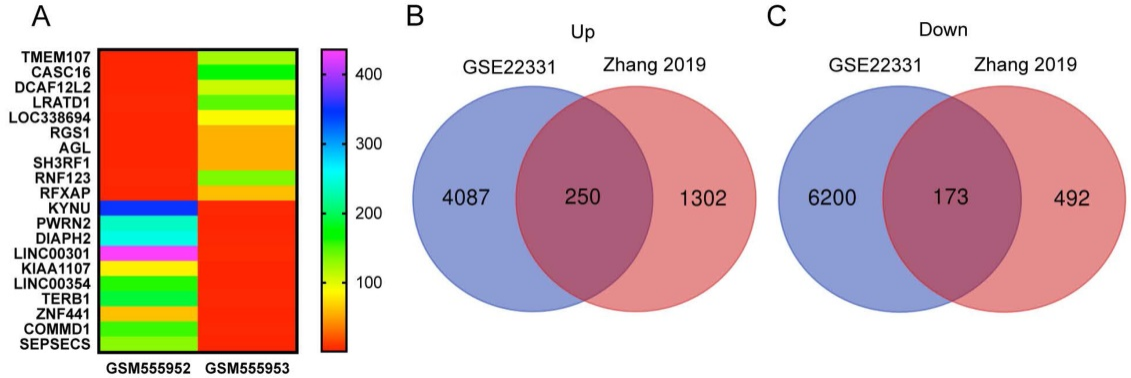
Explore the DEGs between GSE22331 and Xiaoning Zhang's study. (A) Heatmap visualization of the top 10 significantly upregulated and the top 10 significantly downregulated genes in GSE22331. (B-C) DEGs were selected between GSE22331 and Xiaoning Zhang's study. The 2 datasets showed the overlaps of 250 upregulated DEGs and 173 downregulated DEGs. DEGs: differentially expressed genes.

We reanalyzed a publicly available dataset GSE22331, comparing controls to asthenozoospermia, and identified 4337 upregulated and 6373 downregulated genes with the criteria of FC ≥ 2 **(SI Table S5-6)**. The heat map showed the top 10 upregulated and the top 10 downregulated genes in GSE22331 **(Figure 3A)**. Next, we reused the differentially expressed mRNA profile from Xiaoning Zhang's study in 2019 (data was downloaded from Supplementary data Table SIII). Changed upregulation and downregulation genes between asthenozoospermia group and the normal control group with FDR ≤ 0.001 and FC ≥ 2 were considered significant **(SI Table S7-8)**. Then, Venn diagram software was performed to identify DEGs between GSE22331 dataset and Xiaoning Zhang's study. A total of 423 DEGs were detected, including 250 upregulated genes and 173 downregulated genes **(Figure 3B-C)**.

### Gene ontology and pathway enrichment analysis

**Figure 4:**
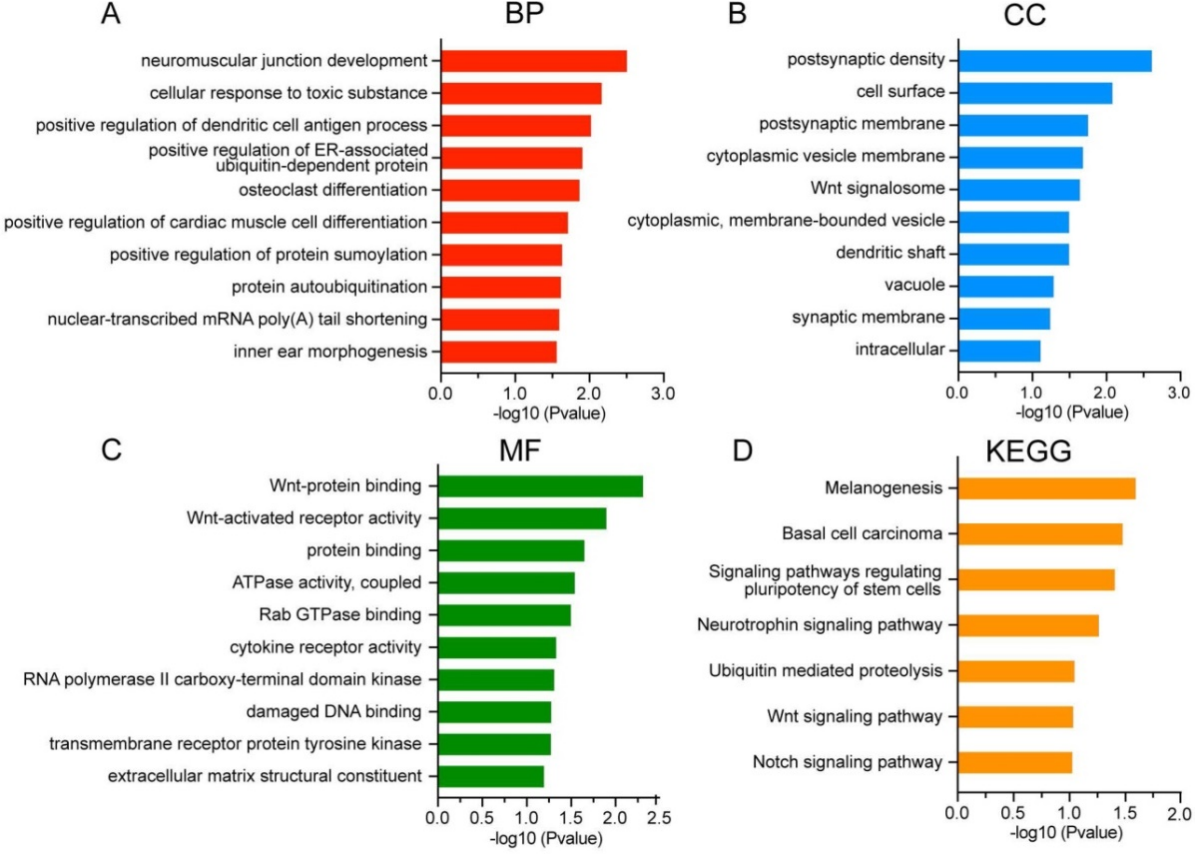
GO and KEGG analysis of DEGs in asthenozoospermia. (A-C) GO analysis of DEGs. (D) KEGG analysis of DEGs. DEGs, differentially expressed genes; BP, biological processes; CC, cellular components; MF, molecular functions.

To further characterize the biological classifications of DEGs, GO terms and KEGG pathways enrichment analysis were performed by DAVID. GO analysis revealed that changes in BP of DEGs were mainly enriched in neuromuscular junction development, cellular response to toxic substance, and positive regulation of dendritic cell antigen process **(Figure 4A)**. Changes in CC of DEGs were significantly enriched in postsynaptic density, cell surface and postsynaptic membrane **(Figure 4B)**. Changes in MF of DEGs were significantly enriched in Wnt-protein binding, Wnt-activated receptor activity and protein binding **(Figure 4C)**. The DEGs were significantly enriched in 7 KEGG pathways including signaling pathways regulating pluripotency of stem cells, Wnt signaling pathway and Notch signaling pathway **(Figure 4D)**.

### PPI network of DEGs and module analysis

**Figure 5:**
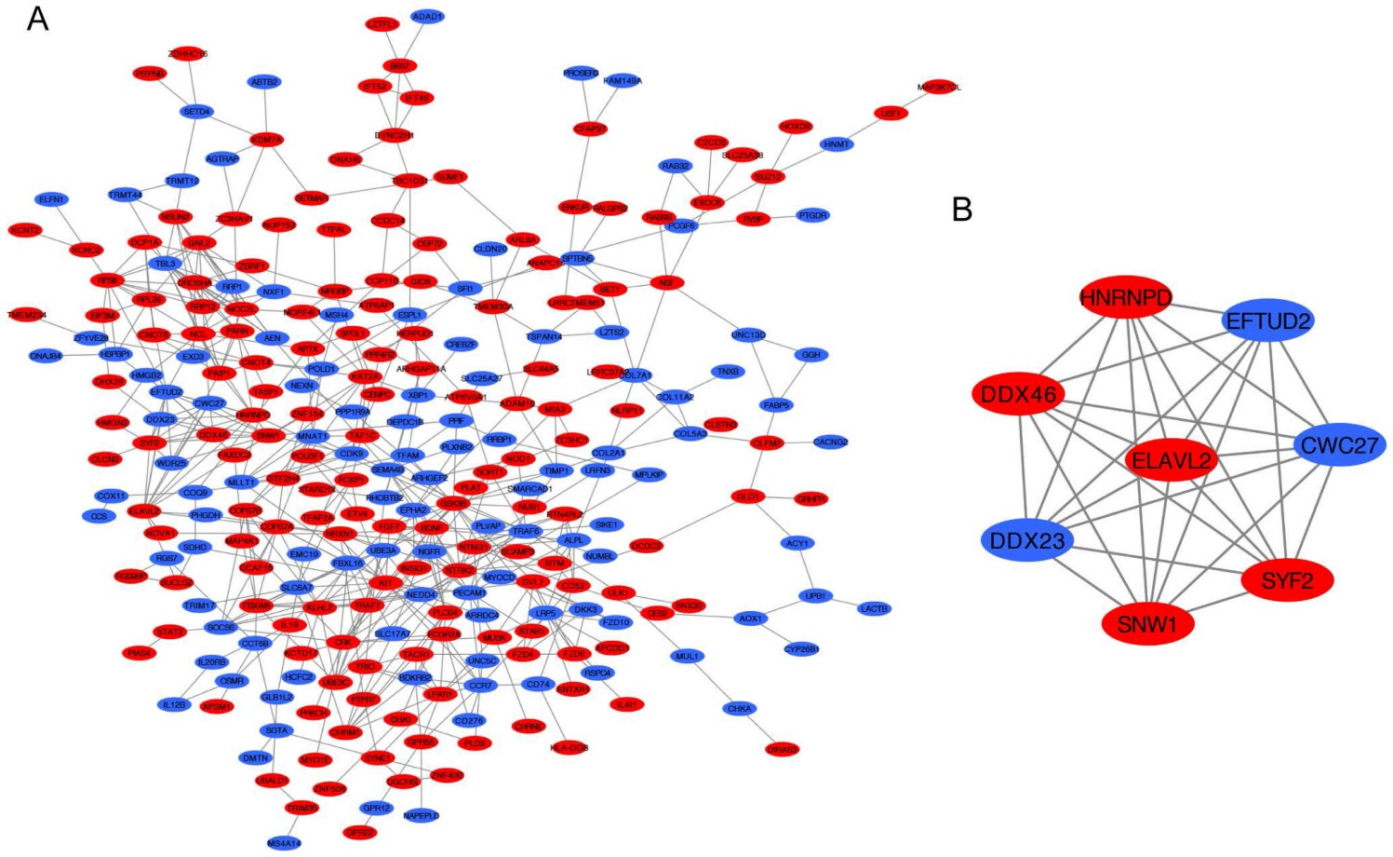
PPI network analysis. (A) Construction of the PPI network. (B) The most significant module was selected from PPI network using the MCODE. Upregulated DEGs were marked in red nodes, and downregulated DEGs were marked in blue nodes. PPI, protein-protein interaction; DEGs: differentially expressed genes.

The PPI network of DEGs was constructed by STRING database including 276 nodes and 550 edges **(Figure 5A)**. Furthermore, the most significant module was established using MCODE which comprised 5 upregulated genes and 3 downregulated genes **(Figure 5B)**.

**Figure 6:**
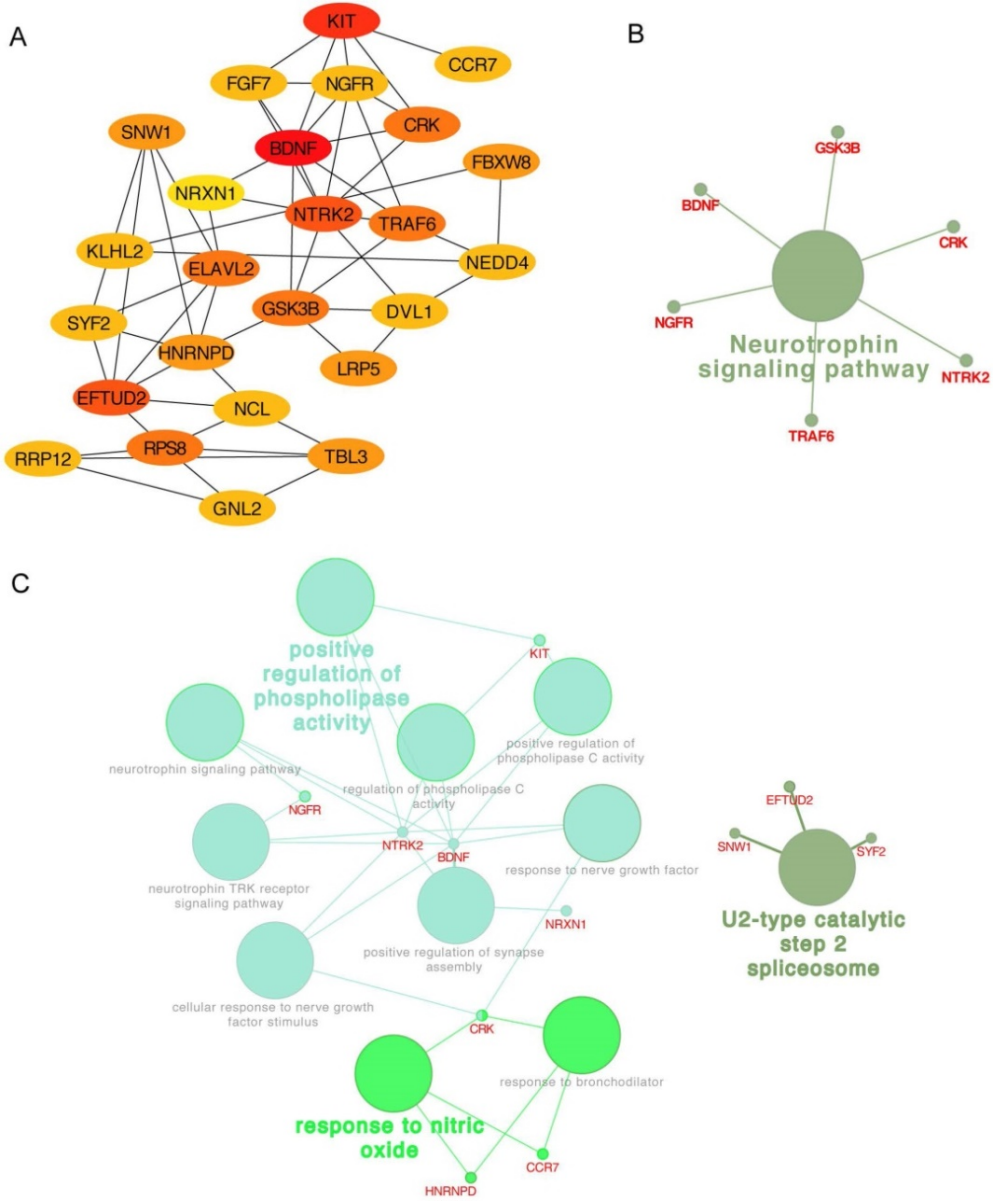
Hub genes analysis. (A) The 25 most important hub genes selected by connectivity degree were screened out using the cytoHubba. The color of node from yellow to red represented the connectivity degree from low to high. (B) The KEGG analysis of hub genes was performed using the ClueGO and the CluePedia. Different color of node denoted different functional annotation of ontology. (C) The GO analysis of hub genes was performed using the ClueGO and the CluePedia. Different color of node denoted different functional annotation of ontology.

In addition, the top 25 genes filtered by connectivity degree in PPI network were identified as hub genes using cytoHubba **(Figure 6A)**, and the most significant gene was BDNF with connectivity degree of 8, followed by NTRK2, HNRNPD, EFTUD2, GSK3B, NGFR, ELAVL2, RPS8, KIT, TRAF6, DVL1, FGF7, SNW1, SYF2, NCL, CRK, NEDD4, TBL3, RRP12, GNL2, NRXN1, KLHL2, LRP5, FBXW8, and CCR7. The pathway enrichment and biological process analysis of hub genes were shown in **Figure 6B-C**. Neurotrophin signaling pathway was in the center position associated with multiple hub genes.

### Construction of miRNA-mRNA network

**Figure 7:**
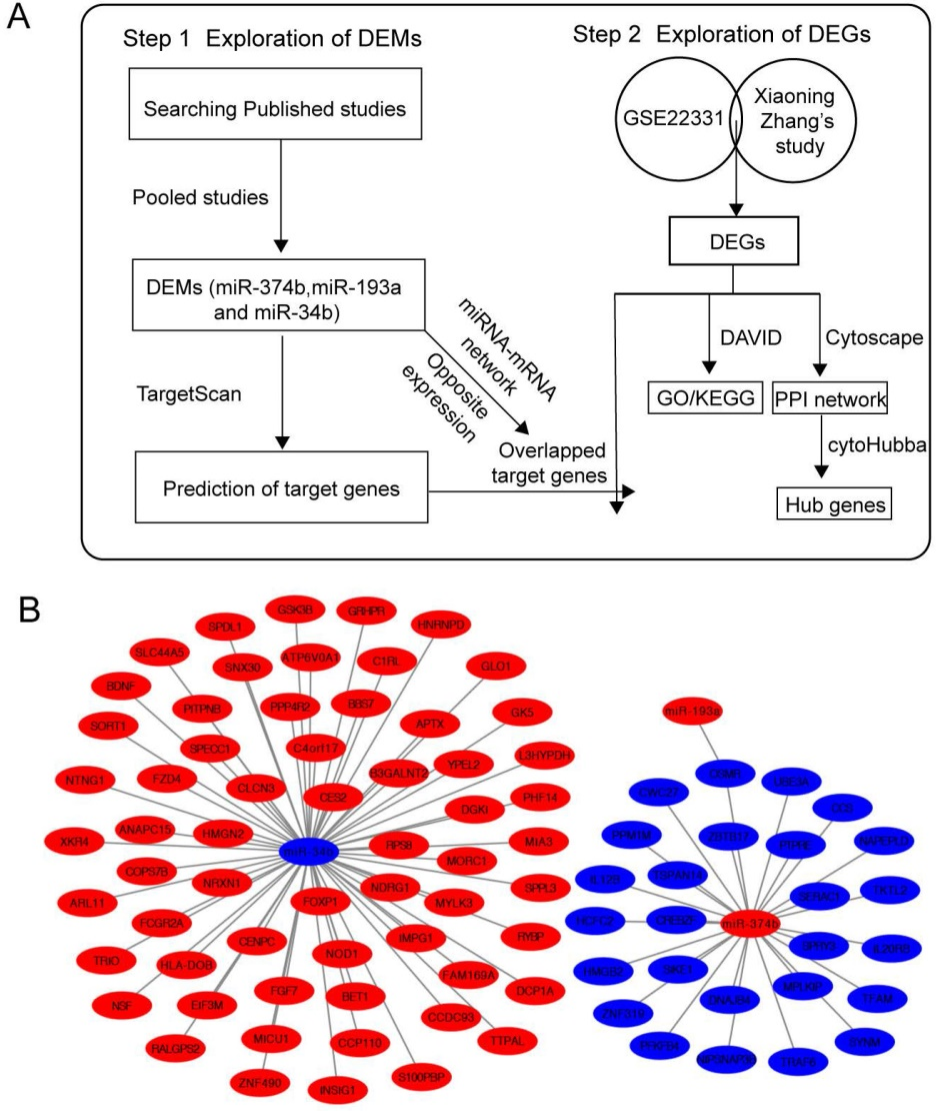
miRNA-mRNA network. (A) Flow chart of network construction process. (B) Network of DEMs and target DEGs. DEMs, differentially expressed miRNAs; DEGs: differentially expressed genes.

Then, we explored the target genes of 3 DEMs using TargetScan database and we obtained the intersection elements between predicted target genes and DGEs. Since the expression of target gene is opposite to its upstream miRNA, we selected the overlapped genes with opposite expressions to their upstream DEMs **(Figure 7A)**. The network of the selected target genes and 3 DEMs was shown in **Figure 7B** using Cystoscape. In this network, miR-193a and miR-374b both negatively regulated OSMR.

### Expression in testicular cancer

**Figure 8:**
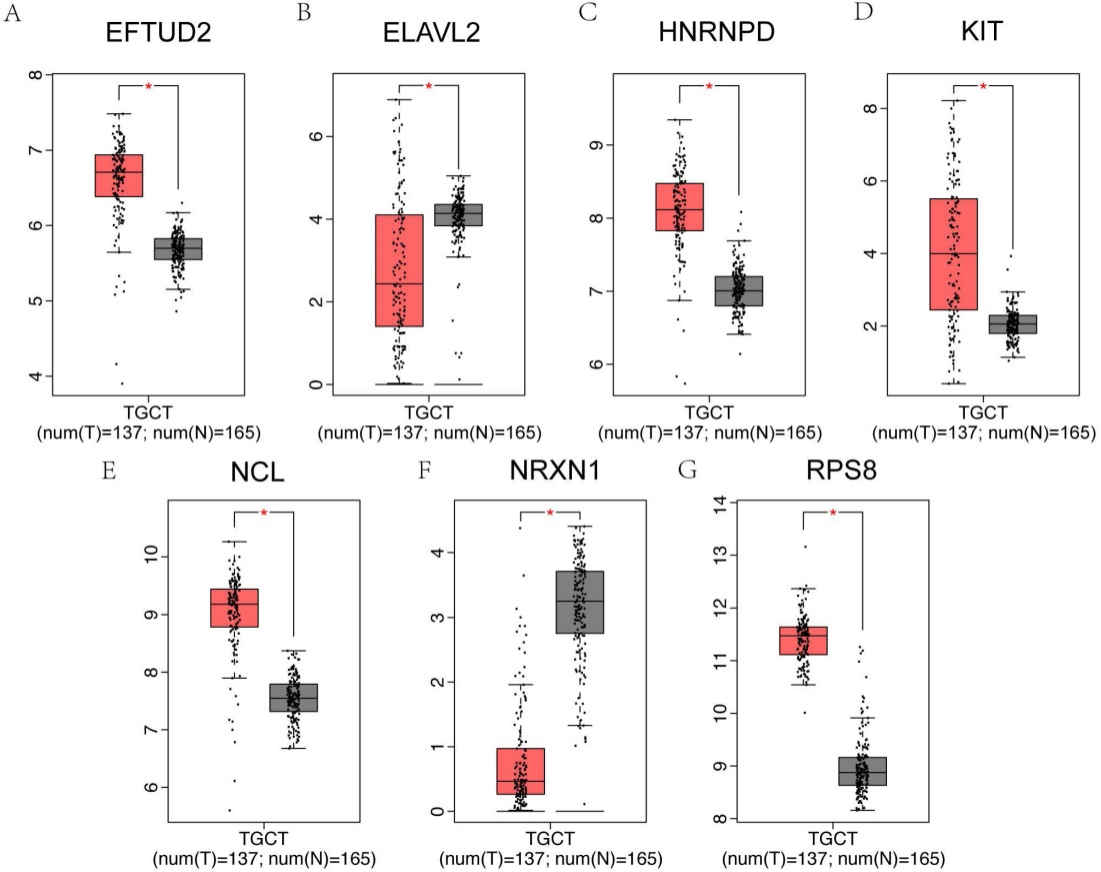
Hub genes expression in testicular cancer. (A-G) The 7 genes of 25 hub genes exhibited significantly different expressions in testicular cancer. EFTUD2, HNRNPD, KIT, NCL and RPS8 were upregulated, ELAVL2 and NRXN1 were downregulated in testicular cancer patients.

It has been reported that patients with testicular cancer might have subnormal sperm motility 27, and we further investigated the alterations of hub genes in testicular cancer patients and normal controls. Among 25 hub genes, EFTUD2, HNRNPD, KIT, NCL and RPS8 were significantly upregulated in testicular cancer patients, however, ELAVL2 and NRXN1 were significantly downregulated using GEPIA online database **(Figure 8A-G)**.

## Discussion

Male infertility is a complicated multifactorial pathological condition affecting around 7% male population 28. Particularly, sperm motility is a crucial factor for fertilization, and over 80% of male infertility is induced by sperm motility impairment 8. Sperm functional or structural defects, deleterious effect of seminal plasma or a combination of these may impair sperm motility 29. Asthenozoospermia, also known as asthenospermia, is a common condition characterized by low sperm motility 30. Interestingly, recent studies have reported that sperm RNAs, including a wide variety of mRNA, miRNA and lncRNA were emerging as critical roles regulating asthenozoospermia 31. Evidence has revealed certain associations between asthenozoospermia and specific variations of sperm transcriptome profile, and also identified some RNA molecules were differentially expressed in asthenozoospermia patients. Our study aimed to investigate significant dysregulated miRNAs and genes in asthenozoospermia and explore the potential regulatory network in asthenozoospermia pathological condition.

First, we identified 3 overlapped DEMs including miR-374b, miR-193a and miR-34b by screening out previous studies. miR-374b seemed to be one of the best normalizing miRNA candidates associated with spermatogenesis and embryogenesis, and it also exhibited a particular behavior and a stable expression in human fertile individuals 32. In addition, decreased miR-374b expression could act as a first indication of increased sperm DNA fragmentation index, indicating that miR-374b would potentially become a diagnostic biomarker of idiopathic infertile males 33. miR-193a was upregulated in sterile triploid fish which might take part in sperm activity and testicular development by targeting functional genes 34. The expression of miR-34b in asthenozoospermia group was significantly lower than control group 20. Besides, the frequency of methylation of the promoter region of miR-34b in infertile men was higher than fertile men, and the highest frequency of methylation was observed in asthenoteratospermia patients 35. These 3 DEMs were proven to be associated spermatogenesis and they were promising for severing as novel biomarkers that could enhance the diagnosis of male infertility. We could further explore the epigenetic regulatory mechanisms of miR-374b, miR-193a and miR-34b in asthenozoospermia.

We next identified 423 DEGs between GSE22331 dataset and Xiaoning Zhang's study. Among these DGEs, 250 genes were upregulated, and 173 genes were downregulated in asthenozoospermia. We observed that DGEs were enriched in development-related pathways, such as signaling pathways regulating pluripotency of stem cells, Wnt signaling pathway and Notch signaling pathway. Studies have reported the germ cell lineage originated in the early stage of development and underwent a series of complicate developmental processes that culminate in the generation of the fully matured gametes, the oocytes and the spermatozoa. Human gametogenesis might be reconstituted from pluripotent stem cells, which would facilitate our understanding of germ cell development and fertilization 36. Another study has indicated that post-transcriptional Wnt signaling could affect spermatozoa through GSK3 through inhibiting protein phosphatase 1 to initiate sperm motility 37. The identification of DEGs and DEMs in asthenozoospermia could provide an insight into the molecular mechanisms driving spermatogenesis and open the possibility of new therapeutic targets to repair impaired sperm physiological function and quality.

Further investigation of PPI network, BDNF, NTRK2, HNRNPD, EFTUD2, GSK3B, NGFR, ELAVL2, RPS8, KIT, TRAF6, DVL1, FGF7, SNW1, SYF2, NCL, CRK, NEDD4, TBL3, RRP12, GNL2, NRXN1, KLHL2, LRP5, FBXW8, and CCR7 were identified as hub genes. The most significant gene was BDNF, a member of the nerve growth factor family. BDNF was detected in the head, neck, and tail of human spermatozoa 38, and exogenous BDNF at 0.133 nM could significantly influence viability and motility of human sperm 39. Interestingly, another neurotrophic factors, NGF, has been reported to rescue Sertoli cell viability 40. NGFR, a low affinity receptor which can bind to NGF, exerted a role in the processes of rat Leydig cell postnatal differentiation and in the regulation of functional activities 41. The expression of miR-4485 was significantly downregulated in the asthenozoospermia patients compared to controls, and KIT, which acted as its target gene, was related to male infertility by bioinformatic analysis 42. A recent study has uncovered a decrease in abundancy of CCR7 receptor in the high-fertile boars 43. In particular, these hub genes were enriched in the neurotrophin signaling pathway, indicating that they were critical for differentiation of nerve cells as well as playing critical roles in the development of reproductive system and the maintenance of spermatozoa normal function. Many aspects of fertility depended on intact neurologic function. Therefore, these similar results provided a comprehensive overview that nerve-related genes might also impact male fertilization, and their relationships required to be investigated further. We also constructed the miRNA-mRNA network, and all of these central interactions suggested us new clues to explore underlying mechanisms between miRNA and its target mRNA in asthenozoospermia.

Testicular cancer is one of the most common cancers in men with major semen parameter disturbances 44, 45. We further tested whether these putative hub genes in asthenozoospermia could extensively affect testicular cancer. We compared the mRNA expression of 25 hub genes between testicular cancer patients and normal controls by GEPIA dataset. As expected, EFTUD2, ELAVL2, HNRNPD, KIT, NCL, NRXN1 and RPS8 were significantly varied in testicular cancer patients.

In our study, the promising miRNAs and mRNA in asthenozoospermia were filtered. They might be dysregulated and exhibited diagnostic values in asthenozoospermia. Additionally, we constructed the miRNA-mRNA network which revealed the underlying mechanism in asthenozoospermia. The development of therapies through direct targeting this network might contribute to the effective treatment of asthenozoospermia. Overall, our findings provided several clues to future diagnosis and clinical treatment of asthenozoospermia.

There were still certain limitations in our current study. There was no available data by searching the GEO database for published microarray results of miRNAs in asthenozoospermia. Therefore, we reused microarray assays data of miRNAs in asthenozoospermia from 4 published studies by applying a systematic search of PubMed and Web of Science. However, these search results might be incomprehensive, and the inclusion criteria of GEMs were different. Additionally, DGEs in GSE22331 only based on FC ≥ 2 without FDR, since there was only one pooled sample in each group. Hence, the repeatability of the results might not be reliable. The findings of the miRNA-mRNA network did not reveal a high degree of several DEMs, because of our strict including criteria of miRNA and mRNA and limited miRNA target prediction database.

## Conclusion

In summary, we were the first to pool published studies and a GEO dataset to identify potential DEMs and DEGs that were involved in asthenozoospermia. Importantly, we also clearly demonstrated the relationship between DEMs and DEGs and suggested regulatory pathways in asthenozoospermia including signaling pathways regulating pluripotency of stem cells, Wnt signaling pathway and Notch signaling pathway. We provided key information to profoundly understand the pathological process of asthenozoospermia and presented theoretical basis to discover novel therapeutic interventions. Further studies *in vivo* and *in vitro* are required to confirm these intriguing mechanistic possibilities.

## Supplementary Material

Supplementary tables.

## Figures and Tables

**Table 1:** Information of 4 studies

Study	Samples	Inclusion criteria of miRNAs
Wang 2011	A = a pool of 58 samples; N = a pool of 100 samples	Copy numbers > 50 and FC > 2
Abu-Halima 2013	A = 9; N = 9	FC > 2 and P value < 0.05
Salas-Huetos 2015	A = 10; N = 10	Significant difference of the mean normCt value between two groups (P value < 0.05)
Heidary 2019	A = 10; N = 10	FC > 2 and P value < 0.05

A: asthenozoospermia; N: normozoospermia; FC: fold change; normCt: normalized threshold cycle.

## Abbreviations

miRNA: microRNA
lncRNA: long noncoding RNA
DEMs: differentially expressed miRNAs
DEGs: differentially expressed genes
BP: biological processes
CC: cellular components
MF: molecular functions
PPI: protein-protein interaction
